# Retraction Note: Aberrant mannosylation profile and FTX/miR-342/ALG3-axis contribute to development of drug resistance in acute myeloid leukemia

**DOI:** 10.1038/s41419-020-2320-8

**Published:** 2020-02-12

**Authors:** Bing Liu, Xiaolu Ma, Qianqian Liu, Yang Xiao, Shimeng Pan, Li Jia

**Affiliations:** 1 College of Laboratory Medicine, Dalian Medical University, Dalian, 116044 Liaoning China; 2 Department of Clinical Laboratory, the First Affiliated Hospital of Dalian Medical University, Dalian, 116011 Liaoning China

**Retraction to: Cell Death & Disease**

10.1038/s41419-018-0706-7 published online 07 June 2018

The Editors-in-Chief have retracted this article because there appears to be replication between a group of data points in the 10.95% quadrant of the U/A (VCR) ALG3 shRNA panel of Fig. 3f and a group of data points in the 4.52% quadrant of the T/A (Paxcitaxel) ALG3 shRNA panel of Fig. 3f. The Editors-in-Chief therefore no longer have confidence in the integrity of the data in Fig. 3. All of the authors disagree with this retraction.

